# Intercalant Aggregation Promotes Nanoscopic Depletion in Droplet Interface Bilayers

**DOI:** 10.1021/acs.jpcb.5c06296

**Published:** 2025-11-14

**Authors:** Caroline Scott, Toshihisa Osaki, Shoji Takeuchi, Sunghee Lee

**Affiliations:** † Department of Chemistry and Biochemistry, 5791 Iona University, 715 North Avenue, New Rochelle, New York 10801, United States; ‡ Artificial Cell Membrane Systems Group, 34762 Kanagawa Institute of Industrial Science and Technology, 3-2-1 Sakado, Takatsu, Kawasaki 213-0012, Japan; § Institute of Industrial Science, 13143 The University of Tokyo, 4-6-1 Komaba, Meguro, Tokyo 153-8505, Japan; ∥ Department of Mechano-Informatics, Graduate School of Information Science and Technology, 13143 The University of Tokyo, 7-3-1 Hongo, Bunkyo, Tokyo 113-8656, Japan

## Abstract

Free volume within lipid bilayers plays a crucial role in determining membrane properties, such as fluidity, permeability, and domain organization, which in turn modulate the functions of membrane proteins and cellular processes. The presence of hydrophobic molecules within bilayers reciprocally influences free volume, and previous studies have shown that solidification of such hydrophobic molecules induces exclusion from the bilayer interior, leading to a reduction in bilayer thickness. Here, we investigate the inclusion and exclusion behaviors of hydrocarbon intercalants by quantifying bilayer thickness. The excluded volume effect expelled the long-chain compound squalane, whereas smaller intercalants, *n*-decane, *p*-xylene, and tetralin, remained incorporated, increasing bilayer thickness. In contrast, crystallizable intercalants, naphthalene and durene, were excluded at concentrations likely to promote aggregation. This exclusion led to a depletion force, resulting in bilayer thinning. Understanding of free volume modulation through membrane composition will not only advance fundamental insights into biological membranes but also guide the further development of synthetic membrane systems.

## Graphical abstract

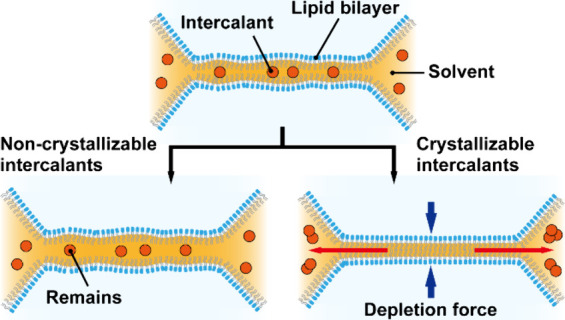

## Introduction

The spontaneous self-assembly of amphiphilic lipid molecules into bilayer structures represents a fundamental principle underlying the architecture of living systems. These bilayer membranes delineate cellular compartments, mediate selective molecular transport, and play critical roles in signal transduction. Although the high packing density of lipid molecules confers robust hydrophobic barrier properties, some free volume remains within the bilayer matrix. This free volume is modulated by the lipid phase (crystalline, gel, fluid, or interdigitated) as well as by various physicochemical factors that promote the formation of voids.^1^ Variations in free volume within the crowded milieu of the lipid bilayer influence key membrane properties, such as fluidity, permeability, and domain organization, which can significantly affect the function of embedded proteins and cellular responses to external stimuli.^2–6^ Cell membranes are crowded environments due to the excluded area interactions among embedded proteins, a phenomenon which influences the conformational transitions of such proteins and in turn, protein function.^7,8^ Despite their fundamental importance, our understanding of crowding phenomena among membrane constituents remains limited.

Given the fundamental role of free volume in governing membrane properties, it is crucial that model membranes accurately recapitulate the physical environment of biological bilayers.^9,10^ Model membranes, such as droplet interface bilayers (DIBs) and planar lipid bilayers, are widely used as cell membrane mimics because they form stable, tunable phospholipid bilayers between aqueous droplets in an oil medium (Figure 1a).^11,12^ DIBs offer a highly controlled platform for assembling and manipulating bilayers with defined lipid compositions and curvature.^13–17^ However, a significant challenge in model systems including DIBs and planar lipid bilayers is the potential for residual hydrocarbon solvent to remain within the bilayer after assembly.^18–21^ The presence of such residual oil can alter the free volume landscape, confounding efforts to study its intrinsic effects on membrane dynamics and permeability. Accordingly, the effects of oil molecules embedded within lipid bilayers have been extensively investigated, as they influence bilayer swelling, domain formation, and line tension, thereby modulating membrane physical properties and phase behavior.^9,22–27^ Atomistic MD simulations of 1,2-dioleoyl-*sn*-glycero-3-phosphocholine (DOPC) bilayers that contain hydrocarbon oil (either hexadecane or squalene) show that for both oils the majority of oil molecules are located in the bilayer midplane (i.e., between the two bilayer leaflets) taking a conformation which is perpendicular to the membrane normal. For hexadecane alone, however, there is also a significant occupancy between the lipid acyl chains.^28^ While a significant recent focus has been the creation of solvent-free model membranes to minimize such complications,^29,30^ studies of oil-containing bilayers remain essential for understanding how hydrophobic molecules impact membrane biophysics.^28,31–33^ Cell membranes naturally encounter a range of oily intercalants, including lipid droplets, which can substantially modulate bilayer structure and properties.^34–37^ Notably, naturally occurring hydrocarbon oil intercalants can alter the organization of membrane components in extremophilic bacteria, highlighting the biological relevance of these interactions.^38,39^ Consequently, elucidating the effects of oil molecules incorporated during bilayer preparation is critical for both accurate interpretation of experimental data and the design of model systems that better mimic native membranes.

**Figure 1:**
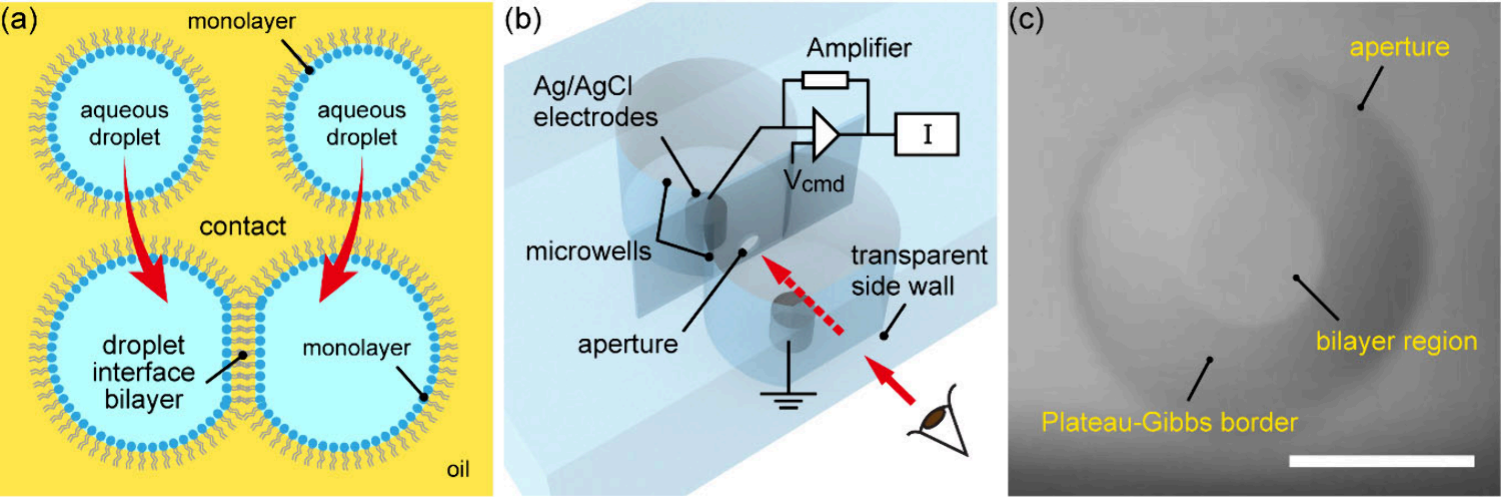
(a) Schematic illustration of a droplet interface bilayer (DIB) formed between a pair of aqueous droplets in lipid-dispersed solvent. (b) Schematic diagram of a custom-fabricated double-well microchip. (c) Representative microscopic image of a DOPC bilayer formed using *n*-hexadecane containing 9 wt % naphthalene. Scale bar: 300 μm.

In seminal work by White, the physical chemistry of planar lipid bilayer membranes was investigated by specific capacitance, revealing how factors such as lipid composition, temperature, and phase state influence the physical properties of the bilayer.^40,41^ Specifically, it was found that the capacitance of the monoolein lipid membrane formed in *n*-hexadecane would abruptly increase as the temperature dropped below the freezing point (*T*_m_ = 18 °C) of hexadecane. This was attributed to the freezing-out of residual hexadecane from the bilayer. This transition reflects the removal of hexadecane below its phase transition temperature from the free volume within the membrane, and this exclusion of hexadecane leads to altered dielectric properties. Intrigued by this clear link between solvent dynamics, free volume, and bilayer properties, we are motivated to further investigate how variations in free volume within lipid bilayers can be driven by an analogous process: an “isothermal solvent freeze-out”.

In the present study, we examine the behavior of six nonpolar hydrocarbon molecules, which behave as intercalants within the intrinsic voids of a planar lipid bilayer, by evaluating their influence on bilayer thickness. These six nonpolar hydrocarbon molecules were delivered to the bilayer in minor quantities as solutes in *n*-hexadecane. The six hydrocarbon intercalants were selected based on their aggregation state at room temperature, with one set potentially being crystallizable and the other set being liquid (Table 1): two crystallizable aromatic hydrocarbon species (naphthalene and durene), two noncrystallizable aromatic hydrocarbon species (tetralin and *p*-xylene), and two noncrystallizable aliphatic hydrocarbons differing in chain length (short-chain compound *n*-decane and long-chain compound squalane). For the planar bilayer system, we employed the DIB configuration, which offers a controllable and well-defined experimental platform for probing bilayer electrical properties.^42,43^ Specific capacitance values were determined from the ionic current response elicited by a square-wave voltage input, and the bilayer thickness was estimated from the capacitance values. We analyzed the inclusion and exclusion behaviors of the hydrocarbon intercalants on the basis of the bilayer thickness changes.

**Table 1:**
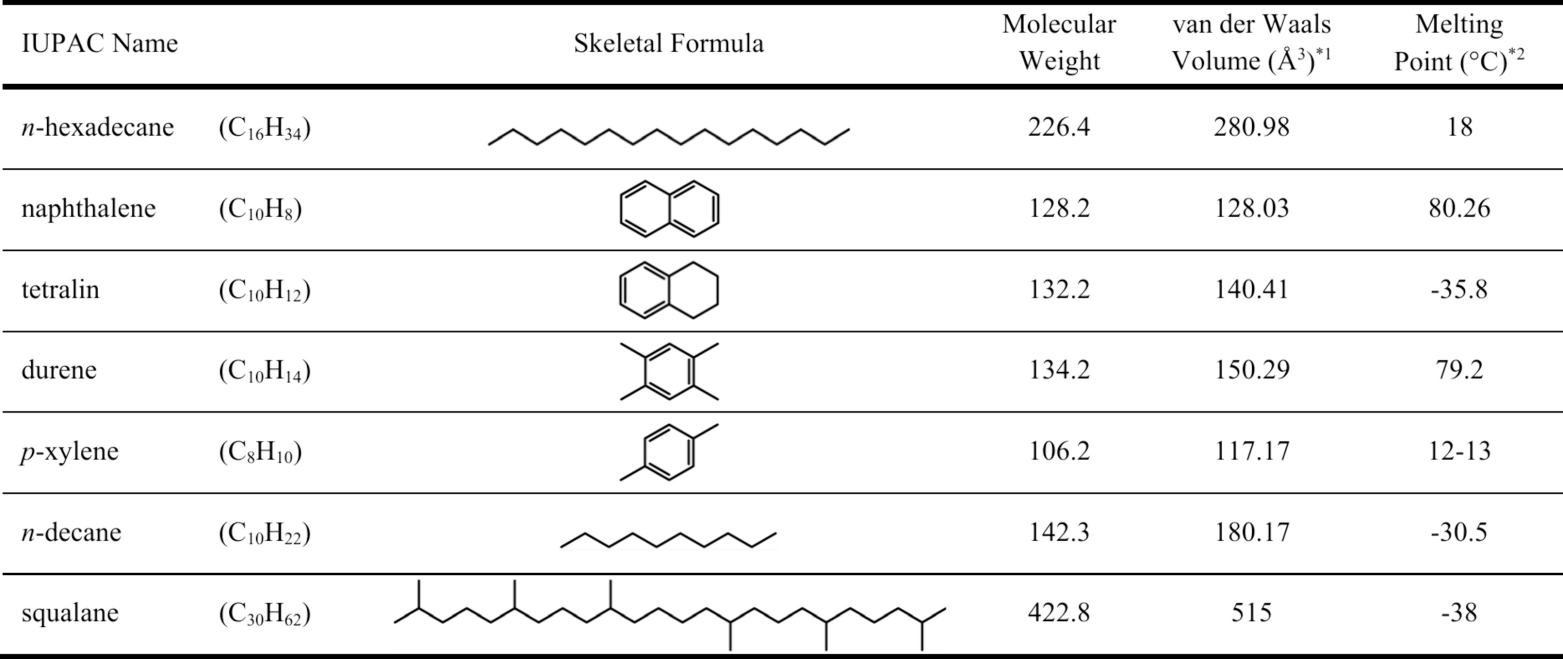
Solvent *n*-Hexadecane and Hydrocarbon Intercalants

*1 www.molinspiration.com. The van der Waals volume reflects the intrinsic size of a molecule, based on the spatial arrangement of its atoms.

*2 NIST Chemistry WebBook, https://webbook.nist.gov/.

## Materials and Methods

### Materials

1,2-Dioleoyl-*sn*-glycero-3-phosphocholine (DOPC) was purchased from Avanti Research (AL, USA). Potassium chloride (0.010 M, Conductivity Standard) was purchased from Thermo Fisher Scientific (MA, USA). The other reagents, including *n*-decane, *p*-xylene, tetralin, *n*-hexadecane, durene, naphthalene, and squalane, were obtained from Sigma-Aldrich (MO, USA). All reagents were used without further purification. Materials for the microchip used in this study were acrylic plates (Acrylite, Mitsubishi Chemical, Japan), silver rods (Nilaco, Japan), and Ag/AgCl paste (BAS, Japan).

### Device Fabrication

A double-well chip previously reported was used for capacitance measurements (Figure 1b).^43^ Briefly, the chip consists of a base part, a perforated separator, a pair of electrodes, and a BNC connector. A computer-aided manufacturing machine (MM-100, Modia Systems, Japan) was used for fabrication of the base part with a 4 mm thick acrylic plate. A pair of microwells, 4 mm diameter and 3 mm depth, was designed on the base part. The separator with a thickness of 75 μm was inserted in the space between the microwells. A 600 μm diameter aperture was opened on the separator. The outside wall of the well was polished for microscopic observation of the bilayer forming at the aperture. A silver rod with 1 mm diameter was embedded at the bottom of each microwell and assembled with a BNC connector. Ag/AgCl paste was applied to the surface of the silver rod for the electrochemical measurements. The chip was thoroughly rinsed with *n*-hexane and ultrapure water and desiccated prior to use.

### Droplet Interface Bilayer (DIB) Formation^42,44^

A desired volume of a phospholipid DOPC dissolved in chloroform was dispensed into a vial and dried under vacuum for more than 2 h. Then, a mixture of *n*-hexadecane and an intercalant in a given weight percent (wt %) was infused in the vial and the DOPC concentration was adjusted to 5 mg/mL. A DIB was formed by sequential injection of the DOPC solution (4 μL) and 0.010 M KCl solution (20 μL) in the microwells of the double-well chip: A water-in-oil droplet is formed in each microwell, and a DOPC monolayer spontaneously forms at the water–oil interface. The monolayers contact each other at the aperture of the separator, resulting in the formation of a DOPC bilayer.

### Capacitance Measurements

The experimental setup for a capacitance measurement is shown in Figure S1.^43^ The double-well chip was connected to a patch-clamp amplifier (Pico2, Tecella, CA, USA), and a digital microscope (YDZ-3F, Yashima Optical, Japan) was horizontally placed to observe the DIB at the aperture through the side wall of the chip. The magnification of the microscope was set to 300. An aluminum-foil cup covered over the chip for a Faraday cage (not shown in Figure S1). The amplifier and the Faraday cage were grounded to suppress electromagnetic noise.

DIB formation at the aperture was observed with the microscope (Figure 1c), and the capacitance change was obtained from the ionic current in response to a square-wave voltage stimulus. The current response shows a steep peak (*I*_max_), followed by charging the membrane capacitance (*C*_m_) with a relaxation time constant (τ). In principle, the membrane capacitance is evaluated by integrating the current response over a time period. In this study, *C*_m_ was estimated by the software from Tecella (CA, USA). Please refer to our previous work for more details.^43^

### Statistical Analysis

For statistical analyses shown in Figures 2 and 3, we applied one-way ANOVA followed by Tukey–Kramer post hoc tests, focusing on the addition of an intercalant. Resultant *p*-values were annotated using the conventional method: “n.s.” (not significant) for *p* > 0.05 and “*” for *p* ≤ 0.01.

## Results and Discussion

To elucidate the structural configuration of the bilayer, we estimated the thickness of its hydrocarbon core based on specific capacitance measurements (Supplementary Text S1). Figure 2a presents the changes in the bilayer thickness following the incorporation of various hydrocarbon intercalants. The data are arranged in order of decreasing bilayer thickness. The corresponding capacitance and thickness values are provided in the Supplementary Tables S1, S2. The control solvent was pure *n*-hexadecane, for which the observed hydrophobic thickness was 4.45 nm for the DOPC bilayer. The inclusion of the short-chain aliphatic hydrocarbon compound, *n*-decane, at 9 wt % led to an increase in bilayer thickness (from 4.45 to 7.56 nm; mean value), whereas the addition of the long-chain aliphatic hydrocarbon compound, squalane, resulted in a marked reduction (from 4.45 to 3.51 nm; mean value). It is well documented that DIBs and other model membranes prepared with hydrocarbon solvents retain a certain amount of solvent within the hydrophobic tail region of the constituent lipids.^9,21,28,45,46^ In agreement with previous studies, shorter-chain aliphatic hydrocarbons are more readily incorporated, thereby increasing the bilayer thickness, an effect reflected in the data shown in Figure 2a. Note that our measured hydrophobic thickness of 4.45 nm for DOPC in *n*-hexadecane is approximately 30% greater than prior values obtained using pressure-controlled thinning in microfluidic devices,^27^ likely because our bilayers are free-floating and not subjected to applied pressure.

**Figure 2:**
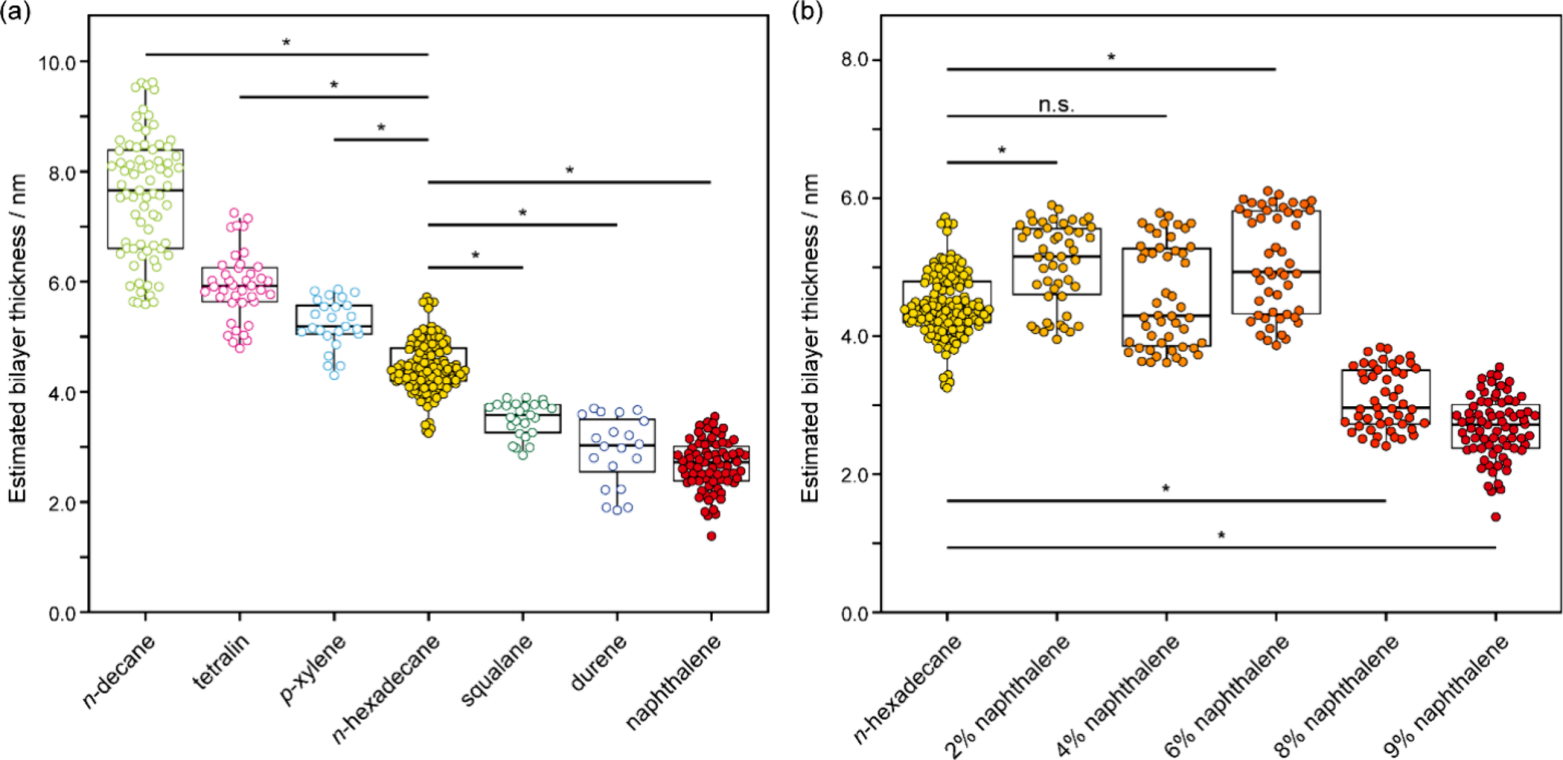
Estimated thicknesses of DIBs formed using *n*-hexadecane as the solvent, with or without the addition of hydrocarbon intercalants listed in Table 1. (a) Intercalants were incorporated at a concentration of 9 wt % relative to the solvent. (b) Naphthalene was added at varying concentrations ranging from 0 to 9 wt % relative to the solvent. For each condition, the number of measurements ranged from 20 to 130. Statistical analysis was performed using one-way ANOVA followed by Tukey–Kramer post hoc tests. Significance levels were indicated as “n.s.” for *p* > 0.05 and “*” for *p* ≤ 0.01. Box plots show the median (center line), interquartile range (box), and full data range, excluding outliers. Detailed values are provided in Supplementary Table S1, S2.

A similar thickening trend as was observed with *n*-decane was seen upon addition of the small noncrystallizable aromatic hydrocarbon compounds tetralin and *p*-xylene: from 4.45 nm for control to 5.93 and 5.24 nm, respectively. Both of these aromatic hydrocarbons possess van der Waals volumes lower than that of hexadecane. This observation further supports the notion that these compounds are sequestered within the hydrophobic region of the bilayer, effectively expanding its hydrocarbon core.

Conversely, the crystallizable aromatic hydrocarbon compounds naphthalene and durene induced a pronounced reduction in bilayer thickness compared to the control condition lacking intercalants (from 4.45 nm for the control to 2.67 nm for naphthalene and 2.94 nm for durene). Despite having van der Waals volumes comparable to tetralin and *p*-xylene and lower than that of hexadecane, naphthalene and durene exhibited contrasting effects to the former.

The formation of a planar lipid bilayer, such as a droplet interface bilayer, can be described in terms of separate stages between the extremes of separated droplets and adherent droplets. As each droplet is initially coated with a DOPC self-assembled monolayer, the mutual approach of droplets can be described as a “zipper-like” process in which a relatively thick film (inclusive of excess oil solvent) transitions into a bimolecular membrane when the tail groups of the respective monolayers appose. First, border suction forces removal of most of the solvent and draws the monolayers together to a position of about 60–100 nm.^47^ Even at this length range, the molar ratio of the respective oil molecules in the bulk solvent mixture (i.e., hexadecane vis-a-vis intercalant) should be reflected in the relatively thick film. Thereafter, van der Waals attraction brings the monolayers even closer (into a range of about 2.5–5 nm), expelling most solvent molecules to a torus or Plateau-Gibbs border in the case of a planar lipid bilayer^48^ or to the bulk region of the oil phase in the case of the droplet bilayer. As this thinning or zipping process progresses, the hexadecane molecule (hexadecane, molecular volume 281 Å^3^) will be progressively excluded due to size effects, leaving the smaller molecules (naphthalene, *p*-xylene, tetralin, durene, or decane) to be incorporated into the bilayer in a concentrated amount (i.e., relative to their bulk molar ratio in hexadecane). For example, naphthalene has a molecular volume of 128 Å^3^ and that of durene is 150 Å^3^. The data presented here is consistent with an envisioned mechanism wherein a concentrated amount of the crystallizable molecules (naphthalene or durene) cannot be accommodated (solvated) within the lipid bilayer without supramolecular aggregates of these molecules being expelled from the bilayer: an isothermal freeze-out.^40^ Further evidence for this concept is provided by tetralin, a compound analogous to naphthalene in chemical structure and molecular volume except that one ring is saturated, and as a result, it is a liquid at room temperature. Similarly, *p*-xylene, an analog compound to durene, has main differences in the number and arrangement of methyl groups attached to the benzene ring. No such exclusion of *p*-xylene or tetralin will occur, owing to their small molecular size and normal liquid state: they will be “dissolved” into the bilayer,^49^ as has been shown in our data in Figure 2a.

To further investigate this hypothesis, we systematically examined the effect of increasing concentrations of naphthalene in *n*-hexadecane on the DIB thickness. As shown in Figure 2b (and Tables S1 and S2), concentrations up to 6% led to a slight increase in membrane thickness compared to those obtained with pure hexadecane (from 4.45 nm in control to 5.04, 4.57, and 5.06 nm for 2%, 4%, and 6%, respectively), consistent with the standard assumption that hydrocarbon intercalant addition increases bilayer thickness (Text S2, Figure S2). However, at higher concentrations of 8% and 9% naphthalene, a significant and abrupt decrease in bilayer thickness was observed (3.08 and 2.67 nm for 8% and 9% naphthalene, respectively). These results suggest that higher concentrations facilitate the aggregation of naphthalene, which is subsequently excluded from the bilayer via an excluded volume mechanism. Intriguingly, the bilayers formed with 8% and 9% naphthalene were even thinner than those formed without any intercalant, implying that not only was naphthalene expelled, but also hexadecane has a lower presence in the bilayer region. We interpret this enhanced thinning as a manifestation of depletion forces, which is an effective attractive force between two leaflets of bilayer, driven by the exclusion of naphthalene; as aggregation progresses, the osmotic pressure difference (bulk solvent relative to bilayer interior) induced by displacement of naphthalene from the bilayer interior promotes stronger contact between droplets. This may also be considered analogous to the depletion-attraction which exists between vesicles in solutions containing large polymer molecules.^50,51^

An alternative hypothesis is that the observed differences in specific capacitance (*C*_p_) arise solely from variations in the bilayer’s dielectric constant (ε). Aromatic hydrocarbons are known to possess higher dielectric constants than alkanes (dielectric constant for naphthalene is 2.372 at 305 K);^52^ thus, a bilayer formed from a mixture of hexadecane and naphthalene would be expected to exhibit a higher dielectric constant, and consequently a higher *C*_p_, than one composed exclusively of hexadecane, given the direct proportionality between *C*_p_ and ε. Importantly, since *C*_p_ is inversely related to bilayer thickness, a higher *C*_p_ would correspond to a thinner bilayer. However, as shown in Figure 2b, our experimental results do not support this expectation, as the mixtures at 6% naphthalene did not result in reduced thickness compared to that of the pure hexadecane bilayer. Additionally, the presence of the aromatic compound *p*-xylene (ε = 2.270 at 293 K)^53^ in hexadecane (Figure 2a) also exhibited reduced capacitance (increased thickness). Therefore, this alternative hypothesis does not account for our observations.

Figure 3 schematically describes the possibilities for positioning of the intercalant within the DOPC bilayer formed by hexadecane as a solvent. If the noncrystallizable aliphatic hydrocarbon intercalant is small and present in low quantities (Figure 3a, upper panel, decane depicted in green oval), it may reside as isolated molecules at a position in the bilayer and contribute to increased membrane thickness. However, if the noncrystallizable aliphatic hydrocarbon intercalant is too large in molecular size or length, such as the case of squalane (Figure 3a, lower panel, elongated dark green), it will be entropically depleted and lead to a decrease in membrane thickness. When a noncrystallizable small aromatic hydrocarbon intercalant is delivered to the bilayer (Figure 3b, upper panel, *p*-xylene or tetralin, depicted by pink squares), these molecules reside as isolated molecules and increase membrane thickness.

**Figure 3:**
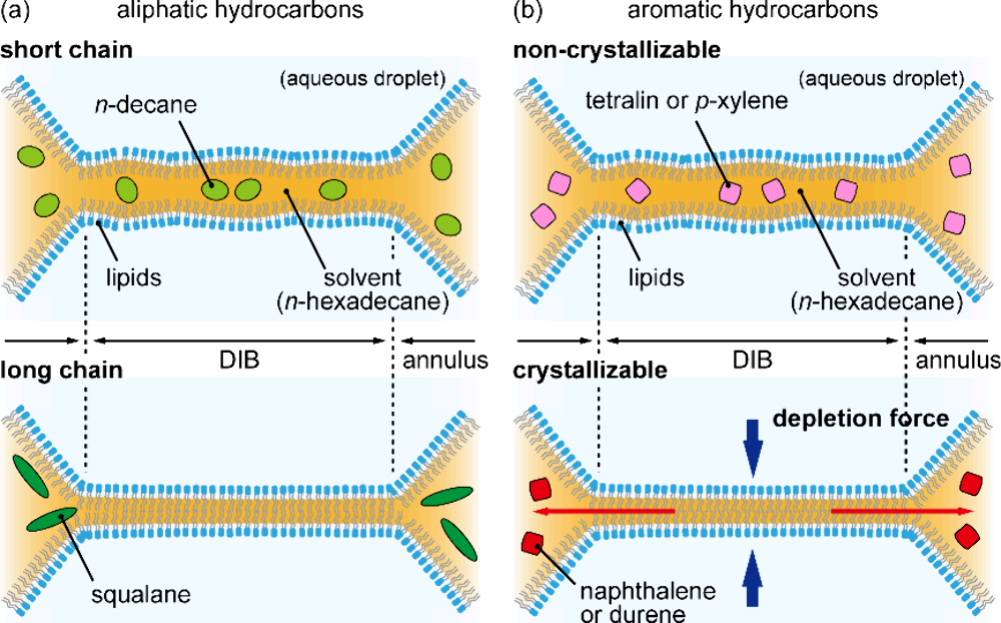
Schematic description of DIBs formed with hexadecane containing (a) aliphatic hydrocarbons or (b) aromatic hydrocarbons.

When a sufficient quantity of a potentially crystallizable small aromatic hydrocarbon intercalant is delivered (Figure 3b, lower panel, naphthalene or durene, depicted by red squares), exclusion of such molecules results in depletion force, leading to thinning of the lipid bilayer. In the control situation, in the absence of intercalants, the equilibrium amount of hexadecane retained within the bilayer is governed by volume exclusion forces operating within the lipid bilayer between the aqueous droplets. However, if naphthalene or durene is present, its crystallizable nature impedes its incorporation into the bilayer core, as these molecules likely undergo self-association, forming supramolecular aggregates that are sterically incompatible with the confined bilayer environment and would have poor solubility in the limited residual hexadecane solvent. Their exclusion gives rise to an osmotic imbalance, manifested as a depletion force, that draws the droplets closer together, thereby expelling intercalants together with the solvent and reducing bilayer thickness (Figure 3b, lower panel).

## Conclusion

Free volume is a critical determinant of membrane-associated processes, including lipid mobility, permeability, and protein function. In this study, we demonstrate that the incorporation of hydrocarbon (oil) molecules into model membranes significantly alters the free volume landscape, leading to measurable changes in bilayer properties such as the membrane thickness, as indicated by capacitance measurements. By systematically varying the molecular characteristics of these hydrocarbon intercalants, we revealed an isothermal freeze-out phenomenon driven by differences in their aggregation behavior. These findings underscore how subtle variations in bilayer composition, particularly in the nature and content of residual oil, can modulate physical properties, with important implications for the interpretation of biophysical measurements in model systems. Beyond refining our understanding of membrane structure and dynamics, this work informs the design of solvent-free biomimetic systems, the development of membrane-based sensors and drug delivery platforms, and efforts to enhance the physiological relevance of in vitro models used to study membrane–protein interactions and transport processes.^54–56^ Ultimately, the ability to control the free volume through lipid and hydrocarbon composition offers a powerful strategy for tailoring membrane function in both biological and synthetic contexts.

## Supplementary Material



## Notes

Overview of the experimental setup; DIB thickness estimation; Capacitance and thickness of DOPC bilayers with varying intercalants; Capacitance and thickness of DOPC bilayers with varying naphthalene concentrations; Theoretical model of bilayer thickness with solvent and intercalants (PDF)
