# Supplementary material for: Intercalant Aggregation Promotes Nanoscopic Depletion in Droplet Interface Bilayers
Source: J Phys Chem B. 2025 Nov 14;129(47):12179–85. doi: 10.1021/acs.jpcb.5c06296 (PMC12670394; doi:10.1021/acs.jpcb.5c06296)
Supplement: Supplementary file 1 [file jp5c06296_si_001.pdf]

Supporting Information

Intercalant Aggregation Promotes Nanoscopic Depletion in Droplet Interface Bilayers

Caroline Scott,[1] Toshihisa Osaki, [2,3,*] Shoji Takeuchi,[2,3,4] and Sunghee Lee [1,*]

Address

1. Department of Chemistry and Biochemistry, Iona University, 715 North Avenue, New Rochelle, NY 10801, USA.
2. Artificial Cell Membrane Systems Group, Kanagawa Institute of Industrial Science and Technology, 3-2-1 Sakado, Takatsu, Kawasaki 213-0012, Japan.
3. Institute of Industrial Science, The University of Tokyo, 4-6-1 Komaba, Meguro, Tokyo 153-8505, Japan.
4. Department of Mechano-Informatics, Graduate School of Information Science and Technology, The University of Tokyo, 7-3-1 Hongo, Bunkyo, Tokyo 113-8656, Japan.

*Corresponding Authors:

S. Lee: SLee@iona.edu

T. Osaki: tosaki@iis.u-tokyo.ac.jp

Supplementary Text S1

DIB Thickness Estimation

The areas of a bilayer, an annulus, and the aperture were evaluated from the microscopic image using ImageJ software (NIH, MD, USA) (see Figure 1c). The Plateau-Gibbs border appearing with the bilayer formation was approximated with a circle by clarifying the image with the CLHAE algorithm (local contrast enhancement) built in the software, and the bilayer area (S_B) inside the border was evaluated. The aperture area (S_0) was also estimated from the image. The annulus area (S_A) was calculated from the difference between S_B and S_0.

The DIB capacitance (C_B) is calculated by subtracting the capacitance of the annulus (C_A) from the membrane capacitance (C_m). Here, we assume that C_A is estimated by multiplying the capacitance before DIB formation (C_0) by the area ratio of the annulus (S_A) to the aperture (S_0). Note that the contribution of C_A is rather small to C_m because the thickness of the annulus is significantly thicker than that of DIB. The DIB thickness is obtained from the equations below.

$$C_B = C_m - C_A \sim C_m - C_0 \cdot S_A / S_0 \qquad \text{(Eq. S1)}$$

$$C_B = \varepsilon_0 \varepsilon_r \cdot S_B / d_B \qquad \text{(Eq. S2)}$$

where ε_0, the vacuum permittivity, ε_r, the dielectric constant of DIB, and d_B, DIB thickness. ε_r was set to 2.1.[1]

Supplementary Figure S1

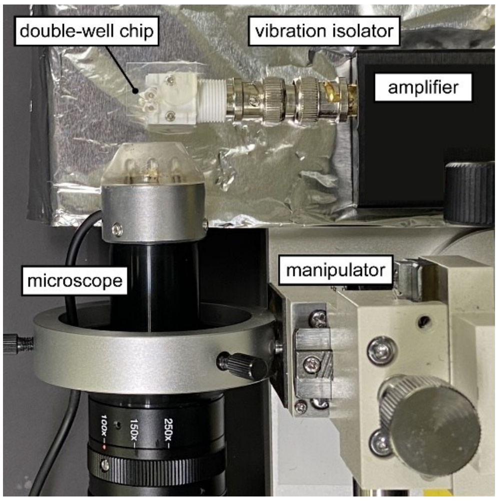

Figure S1. Overview of the experimental setup.

Supplementary Table S1 and S2

Table S1. Capacitance and thickness of DOPC bilayers with varying intercalants at 25 °C.

Intercalant *	Bilayer capacitance / μF/cm²	Estimated bilayer thickness / nm	N **
without intercalant (*n*-hexadecane only)	0.42 ± 0.05	4.45 ± 0.47	130
naphthalene	0.72 ± 0.14	2.67 ± 0.45	75
tetralin	0.32 ± 0.03	5.93 ± 0.62	40
durene	0.67 ± 0.17	2.94 ± 0.63	20
p-xylene	0.36 ± 0.03	5.24 ± 0.45	25
n-decane	0.25 ± 0.04	7.56 ± 1.11	75
squalane	0.53 ± 0.05	3.51 ± 0.32	25

Mean ± standard deviation

* Intercalants were incorporated at a concentration of 9 wt% relative to *n*-hexadecane

** The number of measurements (N) indicates the number of distinct bilayers analyzed

Table S2. Capacitance and thickness of DOPC bilayers with varying naphthalene concentrations.

Intercalant	Bilayer capacitance / μF/cm²	Estimated bilayer thickness / nm	N **
without intercalant (*n*-hexadecane only)	0.42 ± 0.05	4.45 ± 0.47	130
naphthalene 2%	0.37 ± 0.05	5.04 ± 0.60	50
naphthalene 4%	0.42 ± 0.07	4.57 ± 0.75	50
naphthalene 6%	0.38 ± 0.06	5.06 ± 0.74	50
naphthalene 8%	0.62 ± 0.09	3.08 ± 0.44	50
naphthalene 9%	0.72 ± 0.14	2.67 ± 0.45	75

Mean ± standard deviation

** The number of measurements (N) indicates the number of distinct bilayers analyzed

Supplementary Text S2

Theoretical Model of Bilayer Thickness

Building upon the theoretical framework proposed by White,[2] where a planar bilayer membrane is modeled as a thin liquid film composed of monoolein and *n*-hexadecane, we extend this model to encompass a ternary system comprising a phospholipid (DOPC), a solvent (*n*-hexadecane), and an intercalant (e.g., naphthalene). In this extended model, the apparent bilayer thickness is expressed as the cumulative contribution of each component, calculated by dividing its surface density by its volume density.

$$\delta_{bilayer} = \frac{\sigma_{PC}}{N_A} \cdot \frac{M_{PC}}{\rho_{PC}} + \frac{\sigma_S}{N_A} \cdot \frac{M_S}{\rho_S} + \frac{\sigma_I}{N_A} \cdot \frac{M_I}{\rho_I} \quad \text{(Eq. S3)}$$

Here, σ denotes the surface density [/cm^2], M the molecular weight [g/mol], ρ the volume density [g/cm^3], and N_A Avogadro's number [/mol]. The subscripts PC, S, and I correspond to DOPC, *n*-hexadecane, and the intercalant, respectively. The parameters used in this model are summarized in Table S3. Due to the limited solubility of the intercalant in the solvent, we assumed its volume density to be equal to that of *n*-hexadecane. The surface densities of *n*-hexadecane and the intercalant were allowed to vary with their respective interactions with the hydrophobic tails of DOPC or their concentrations, whereas the surface density of DOPC was considered constant, based on its ability to form stable bilayers under all tested conditions. In accordance with previous work,[5] the hydrophobic region of DOPC was modeled as a pair of 1-heptadecene molecules. The adopted surface density of 1-heptadecene (5 nm^{-2}) is consistent with twice the value derived from the surface area of typical phospholipids (0.7 nm^2).[3]

Using the extended model, we estimated bilayer thickness under two distinct assumptions regarding surface densities. In the first scenario, the surface densities of DOPC and *n*-hexadecane were assumed to remain constant, while the surface density of naphthalene was considered to increase proportionally with its added concentration. This assumption reflects a typical planar bilayer containing residual solvent, wherein additional naphthalene is retained within the bilayer region in proportion to the amount introduced. Under this condition, a slight increase in bilayer thickness was observed upon the addition of naphthalene, as shown in Figure S2. This modest effect is attributed to the relatively low concentration of naphthalene in the bilayer compared to that of the solvent (no more than 9 wt%). To account for the more pronounced thickening observed with intercalants such as *n*-decane and tetralin, the model would require the assumption that these compounds are enriched within the bilayer to a degree exceeding their added concentrations.

In the second scenario, we assumed a surface density of zero for naphthalene, indicating its complete exclusion from the bilayer, and varied the surface density of *n*-hexadecane. However, under this condition, the model failed to replicate the experimentally observed reduction in bilayer thickness compared to the bilayer formed with *n*-hexadecane alone (i.e., without intercalants). To account for such thinning, it was necessary to postulate that *n*-hexadecane is also excluded from the bilayer region alongside naphthalene. This modeling result supports the hypothesis that a "freeze-out" phenomenon occurs in the presence of naphthalene, a crystallizable intercalant. The exclusion of naphthalene appears to trigger a depletion force, which in turn facilitates the co-

exclusion of *n*-hexadecane from the bilayer interior, resulting in the observed membrane thinning.

Table S3. Parameters for theoretical model [2]

parameters	DOPC (1-heptadecene) *	*n*-hexadecane	naphthalene
surface density σ [/cm^2]	$5 \cdot 10^{14}$	-	-
molecular weight M [g/mol]	239	226	128
volume density ρ [g/cm^3]	0.789	0.774	0.774 **

* The parameters of 1-heptadecene were used for the hydrophobic region of DOPC.

** The value of *n*-hexadecane was used for that of naphthalene.

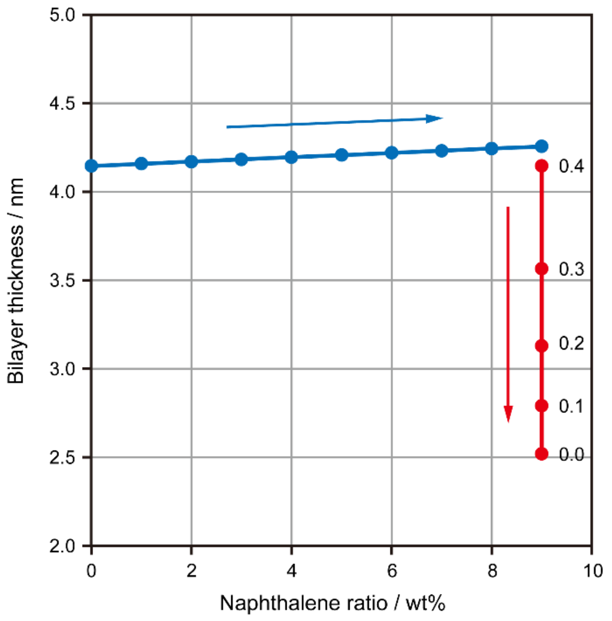

Figure S2. Estimated bilayer thickness based on the extended model. The blue plot represents the first scenario, in which bilayer thickness slightly increases with the increase in naphthalene concentration, assuming constant surface densities for DOPC and *n*-hexadecane. The red plot represents the second scenario, showing the influence of residual solvent on bilayer thickness. Numerical labels indicate the ratio of the surface density of *n*-hexadecane to that of DOPC.

References

(1) Plant, A. L.; Gueguetchkeri, M.; Yap, W. Supported Phospholipid/Alkanethiol Biomimetic Membranes: Insulating Properties. *Biophys. J.* **1994**, *67* (3), 1126–1133. https://doi.org/10.1016/S0006-3495(94)80579-X.

(2) White, S. H. Temperature-Dependent Structural Changes in Planar Bilayer Membranes: Solvent "Freeze-Out." *Biochim. Biophys. Acta - Biomembr.* **1974**, *356* (1), 8–16. https://doi.org/10.1016/0005-2736(74)90289-2.

(3) Zhuang, X.; Makover, J. R.; Im, W.; Klauda, J. B. A Systematic Molecular Dynamics Simulation Study of Temperature Dependent Bilayer Structural Properties. *Biochim. Biophys. Acta - Biomembr.* **2014**, *1838* (10), 2520–2529. https://doi.org/10.1016/j.bbamem.2014.06.010.
